# Ghrelin Treatment of Cachectic Patients with Chronic Obstructive Pulmonary Disease: A Multicenter, Randomized, Double-Blind, Placebo-Controlled Trial

**DOI:** 10.1371/journal.pone.0035708

**Published:** 2012-05-01

**Authors:** Keisuke Miki, Ryoji Maekura, Noritoshi Nagaya, Masamitsu Nakazato, Hiroshi Kimura, Shinsuke Murakami, Shunsuke Ohnishi, Toru Hiraga, Mari Miki, Seigo Kitada, Kenji Yoshimura, Yoshitaka Tateishi, Yasuji Arimura, Nobuhiro Matsumoto, Masanori Yoshikawa, Kenichi Yamahara, Kenji Kangawa

**Affiliations:** 1 Department of Internal Medicine, National Hospital Organization Toneyama National Hospital, Toyonaka, Japan; 2 Department of Regenerative Medicine, National Cerebral and Cardiovascular Center Research Institute, Suita, Japan; 3 Department of Neurology, Respirology, Endocrinology and Metabolism, Internal Medicine, Faculty of Medicine, University of Miyazaki, Miyazaki, Japan; 4 Second Department of Internal Medicine, Nara Medical University, Kashihara, Japan; 5 Department of Biochemistry, National Cerebral and Cardiovascular Center Research Institute, Suita, Japan; Leiden University Medical Center, The Netherlands

## Abstract

**Background:**

Pulmonary cachexia is common in advanced chronic obstructive pulmonary disease (COPD), culminating in exercise intolerance and a poor prognosis. Ghrelin is a novel growth hormone (GH)-releasing peptide with GH-independent effects. The efficacy and safety of adding ghrelin to pulmonary rehabilitation (PR) in cachectic COPD patients were investigated.

**Methodology/Principal Findings:**

In a multicenter, randomized, double-blind, placebo-controlled trial, 33 cachectic COPD patients were randomly assigned PR with intravenous ghrelin (2 µg/kg) or placebo twice daily for 3 weeks in hospital. The primary outcomes were changes in 6-min walk distance (6-MWD) and the St. George Respiratory Questionnaire (SGRQ) score. Secondary outcomes included changes in the Medical Research Council (MRC) scale, and respiratory muscle strength. At pre-treatment, serum GH levels were increased from baseline levels by a single dose of ghrelin (mean change, +46.5 ng/ml; between-group p<0.0001), the effect of which continued during the 3-week treatment. In the ghrelin group, the mean change from pre-treatment in 6-MWD was improved at Week 3 (+40 m, within-group p = 0.033) and was maintained at Week 7 (+47 m, within-group p = 0.017), although the difference between ghrelin and placebo was not significant. At Week 7, the mean changes in SGRQ symptoms (between-group p = 0.026), in MRC (between-group p = 0.030), and in maximal expiratory pressure (MEP; between-group p = 0.015) were better in the ghrelin group than in the placebo group. Additionally, repeated-measures analysis of variance (ANOVA) indicated significant time course effects of ghrelin versus placebo in SGRQ symptoms (p = 0.049) and MEP (p = 0.021). Ghrelin treatment was well tolerated.

**Conclusions/Significance:**

In cachectic COPD patients, with the safety profile, ghrelin administration provided improvements in symptoms and respiratory strength, despite the lack of a significant between-group difference in 6-MWD.

**Trial Registration:**

UMIN Clinical Trial Registry C000000061

## Introduction

Pulmonary cachexia is common in the advanced stage of chronic obstructive pulmonary disease (COPD), and it is an independent risk factor for death in such patients [1], [2]. Based on the notion that advanced COPD affects the whole body and causes wasting syndromes, many different therapeutic approaches have been attempted to improve this syndrome [1], [3].

Pulmonary rehabilitation (PR) including exercise training is well accepted to improve exercise performance and quality of life in COPD patients [4], and it has been regarded as a nutritional adjunct therapy [5].

During the 1970s and 1980s, many gut peptides were identified [6]. Ghrelin, first discovered in 1999 as a novel growth hormone (GH)-releasing peptide isolated from the stomach, has been identified as an endogenous ligand for GH secretagogue receptor [7]. Ghrelin also has a variety of GH-independent effects, such as causing a positive energy balance and weight gain by decreasing fat utilization [8], stimulating food intake [9], and inhibiting sympathetic nerve activity [10], [11]. In addition, plasma ghrelin levels were elevated in cachectic COPD patients and were associated with the cachectic state and pulmonary function abnormalities, suggesting that endogenous ghrelin increased to compensate for the cachectic state and may provide important clues to improve the catabolic-anabolic imbalance in such patients[12]. In an open-label pilot study, we showed that ghrelin treatment increased walking distance in cachectic COPD patients [13]. Based on the above available evidence, a multicenter, randomized, double-blind, placebo-controlled study was conducted to test the hypothesis that the addition of ghrelin treatment to PR might benefit cachectic COPD patients. The objectives were to investigate the efficacy and safety of adding ghrelin to PR in cachectic COPD patients.

**Figure 1:**
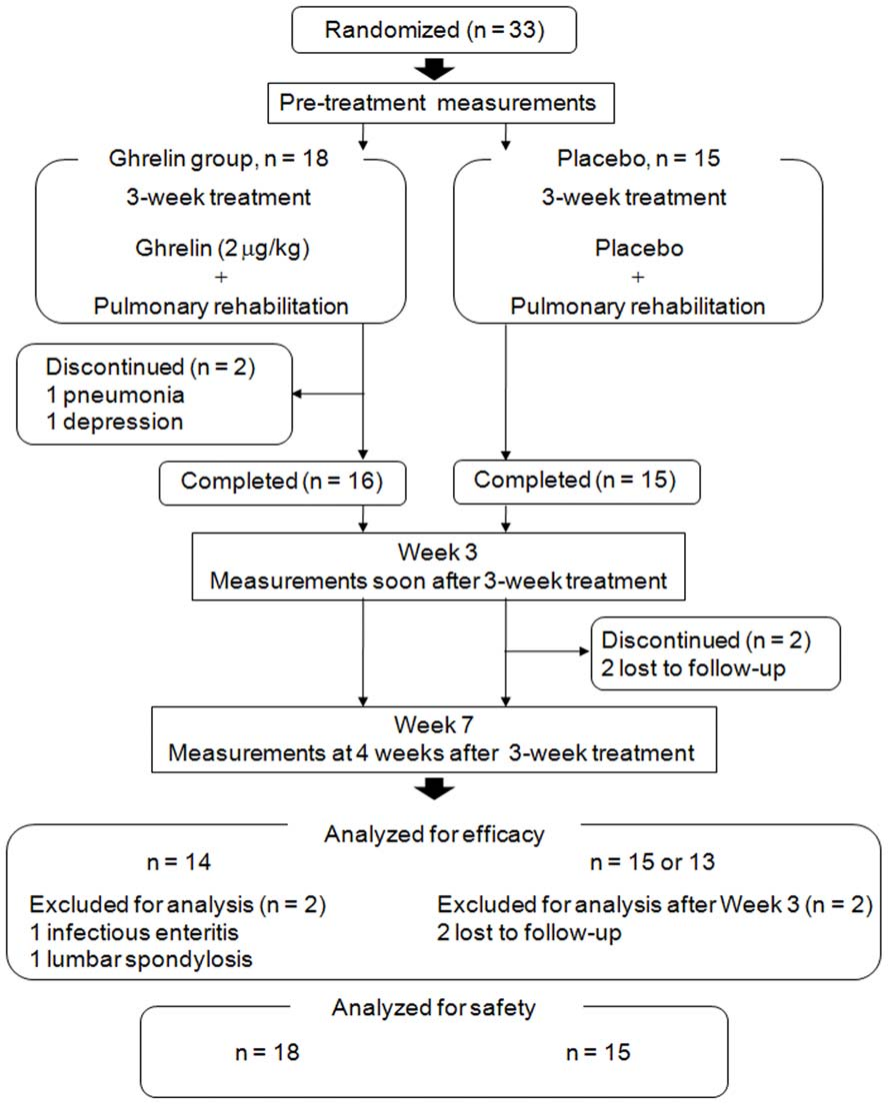
Trial profile.

## Methods

The protocol for this trial, supporting CONSORT checklist, and Supplementary Methods are available as supporting information; see Protocol S1, Checklist S1, and Supplementary Methods S1.

### Study Design and Patients

The study was a 3-week, multicenter, randomized, double-blind, placebo-controlled trial of ghrelin administration during PR. The study was finally conducted at four clinical centers (National Cerebral and Cardiovascular Center, Miyazaki University School of Medicine, Nara Medical University, and National Hospital Organization Toneyama National Hospital) in Japan from September 2005 through May 2009, because Graduate School of Medicine, Osaka City University did not participate just before the start of the clinical trial. The study was conducted according to the Declaration of Helsinki and Good Clinical Practice guidelines and approved by the ethics committees of all participating study centers: The ethics committee of the National Cerebral and Cardiovascular Center (approval number, M17–13); The ethics committee of Miyazaki University School of Medicine (approval number, 218): The ethics committee of Nara Medical University (approval number, 05–012); and The ethics committee of the National Hospital Organization Toneyama National Hospital (approval number, 0311). All patients gave written informed consent (in Japanese). The inclusion criteria were as follows: 1) severe to very severe COPD (forced expiratory volume in one second (FEV1)/forced vital capacity (FVC) of less than 70% and FEV1 percent predicted of less than 50%); 2) underweight (body mass index (BMI)<21 kg/m^2^); 3) clinically stable and able to participate in PR; 4) between 20 and 85 years old; and 5) signed the agreement for participation in this study. Participants were excluded for any of the following: 1) malignant tumors; 2) active infection; 3) severe heart disease; 4) hepatic dysfunction (serum aspartate aminotransferase and alanine aminotransferase levels at least twice the upper limit of normal); 5) renal dysfunction (serum creatinine levels ≥2.0 mg/dl); 6) asthma; 7) definitely or possibly pregnant; 8) change in drug regimen within 4 weeks before participation in this study; or 9) judged to be unable to participate in this study by their physician. This study was registered with UMIN (University Hospital Medical Information Network in Japan: http://www.umin.ac.jp/ctr/), number C000000061.

**Table 1:** Patients’ baseline characteristics. *

	Ghrelin, n = 14	Placebo, n = 15	p value
Age, years†	70.5 (6.2), 63–80	73.9 (6.0), 63–82	0.15
Sex, male/female‡	13/1	13/2	1.00
BMI, kg/m^2^ †	18.6 (2.1), 14.4–20.9	18.0 (2.1), 14.7–20.9	0.38
Cigarette smoking, pack years†	62.0 (30.9), 3.8–125	52.5 (28.8), 0.0–97.5	0.38
Pulmonary function†			
FEV1, L	0.78 (0.20), 0.54–1.21	0.77 (0.21), 0.47–1.21	0.90
%FEV1, % predicted	31.6 (8.1), 21.2–49.5	34.5 (9.1), 17.7–45.9	0.32
FEV1/FVC, %	38.0 (8.9), 24.6–50.5	38.8 (8.7), 25.4–52.9	0.74
VC, L	2.48 (0.37), 1.90–3.45	2.52 (0.50), 1.62–3.69	0.98
%VC, %	78.8 (9.3), 64.0–94.3	84.5 (12.6), 71.4–113.4	0.38
Exercise capacity on ICPET†			
Peak 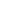 , ml/kg/min	11.5 (3.3), 5.2–17.5	11.3 (3.5), 6.2–18.7	0.74
6-MWD, m†	328 (110), 148–619	315 (118), 85–498	0.84
SGRQ†			
Total score	58.2 (16.5), 36.3–84.4	50.2 (15.5), 21.3–77.3	0.23
Symptoms score	61.5 (22.5), 29.4–97.5	51.6 (19.8), 19.7–78.5	0.34
Activity score	72.5 (14.9), 41.7–92.5	65.9 (16.3), 35.3–92.5	0.34
Impacts score	46.7 (19.5), 20.0–84.4	39.2 (17.7), 9.4–69.7	0.53
Medications‡			
LAMA	9	6	0.27
SAMA	3	2	0.65
LABA	9	7	0.46
SABA	2	0	0.22
ICS	5	2	0.21
Methylxanthines	7	7	1.00

Data are presented as means (SD), and the minimum and maximum values unless otherwise stated. BMI = body mass index; FEV_1_ = forced expiratory volume in one second; FVC = forced vital capacity; ICPET = incremental cardiopulmonary exercise testing; ICS = inhaled corticosteroids; LABA = long-acting β_2_-agonist; LAMA = long-acting muscarinic antagonist; SABA = short-acting β_2_-agonist; SAMA = short-acting muscarinic antagonist; VC = vital capacity.

* The groups shown represent only patients analyzed for efficacy. Medications are not mutually exclusive, and data are presented separately.

† Analyzed using a Wilcoxon rank sum test.

‡ Analyzed using a Fisher’s exact test.

### Randomization and Interventions

Randomization was done in each center considered as a block. The randomization list was generated by a statistician from Hamamatsu University School of Medicine and maintained there until the study was finished and unblinded. Neither the physicians nor the patients were aware of the treatment assignments. Patients who met the eligibility criteria were enrolled and randomly assigned in a 1∶1 ratio to receive PR with either ghrelin (2 µg/kg) or placebo twice a day for 3 weeks in hospital. The administration of ghrelin (2 µg/kg, ghrelin solution with 10 ml saline) or placebo was done intravenously over 30 minutes at a constant rate and repeated twice a day for 3 weeks. Patients were tested at pre-treatment, Week 3 after start of ghrelin or placebo administration with PR, and Week 7 after start of ghrelin or placebo administration with PR, i.e., 4 weeks after the completion of the combination treatment (Figure 1).

### Preparation of Human Ghrelin

Human ghrelin obtained from the Peptide Institute Inc. was dissolved in distilled water with 3.75% D-mannitol and sterilized as described previously [13]. Ghrelin was stored in 2-ml volumes, each containing 120 µg ghrelin. The chemical nature and content of the human ghrelin in vials were rarefied as described previously [13]. All vials were stored frozen at −30°C until the time of preparation for administration.

### Pulmonary Rehabilitation

Exercise training, which was included in the PR program, was conducted in three sets daily, every weekday for 3 weeks (i.e. 15 days) at high-intensity^ ^targets. Additional details are described online in Supplementary Methods S1.

### Outcome Measure

Efficacy: The primary outcomes were changes in 6-min walk distance (6-MWD) and the score evaluated using the St. George Respiratory Questionnaire (SGRQ) [14]. Secondary outcomes were changes in the health-related QoL (HRQoL) score using the Short-Form 36 questionnaire (SF 36 v2™ Health Survey, Japanese version) [15], [16], [17] and the Medical Research Council (MRC) dyspnea scale [18], peak oxygen uptake (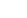), food intake, FEV1/FVC, vital capacity (VC), respiratory muscle strength, and plasma norepinephrine levels in the resting condition.

Safety: All randomized patients who received at least one dose of the study treatments (ghrelin group, n = 18; placebo group, n = 15) were included in the safety analyses using intention-to-treat analysis. Blood tests were done up to Week 7. All serious adverse events were monitored throughout the study period.

**Figure 2:**
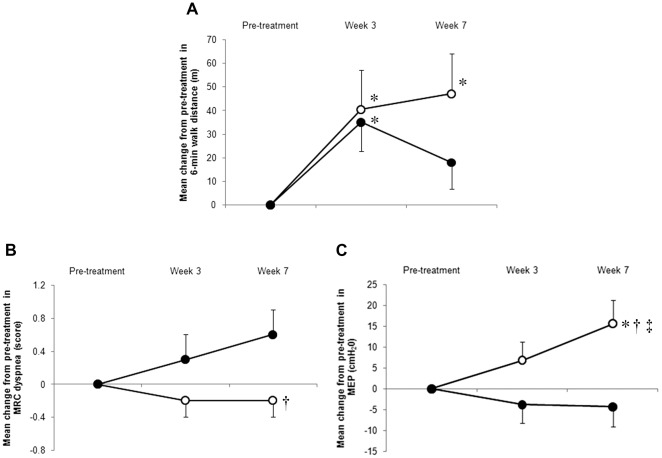
Change from pre-treatment in 6-min walk distance (6-MWD), Medical Research Council (MRC) score, and maximal expiratory pressure (MEP) over time. Open circles, ghrelin; closed circles, placebo. Data are presented as mean differences±SE. * p<0.05: change between pre- and post-treatment (within-group difference). ^†^ p<0.05: change between pre-treatment and post-treatment (between ghrelin and placebo group difference). ^‡^ p<0.05: time course effect of ghrelin versus placebo by repeated-measures ANOVA. A) In both groups, 6-MWD increases significantly to a similar level from pre-treatment at Week 3. Prolonged effects can be seen in the ghrelin group at Week 7, though the improvement in 6-MWD declined in the placebo group. B) Though the MRC score became progressively worse in the placebo group, the maintained effects in the MRC score can be seen in the ghrelin group at Week 7. C) Repeated-measures ANOVA indicated significant time course effects of ghrelin versus placebo in MEP (F (2, 51) = 4.17, p = 0.021).

### 6-min Walk Test

The 6-MWD was measured as described previously [13].

### Cardiopulmonary Exercise Testing (CPET)

While breathing room air with a mask, symptom-limited CPET was conducted on an electrically braked cycle ergometer using an incremental protocol (continuous ramp rate of 5 W/min). Expired gas data were measured breath-by-breath and collected as 30-s averages at rest and during exercise. The CPET was done until subject exhaustion.

**Table 2:** Changes in pre-treatment exercise capacity, pulmonary function and other parameters during pulmonary rehabilitation with ghrelin or placebo.

	At Week 3			At Week 7		
	Ghrelin,n = 14	Placebo,n = 15	Treatment effect(95% CI; p value)	Ghrelin,n = 14	Placebo,n = 13	Treatment effect(95% CI; p value)
Exercise capacity						
6-MWD, m	40 (17)*	35 (12)*	5 (−37 to 48; 0.81)	47 (17)*	18 (11)	29 (−15 to 73; 0.19)
Peak 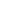 ,ml/min/kg	1.2 (0.4)*	0.5 (0.3)	0.7 (−0.4 to 1.8; 0.21)	ND	ND	ND
Peak 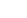 /HR,ml/beats	0.5 (0.2)*	−0.4 (0.5)	0.9 (−0.2 to 2.0; 0.11)	ND	ND	ND
PFT						
FEV1/FVC, %	−1.1 (1.0)	−2.7 (0.9)*	1.6 (−1.2 to 4.3; 0.26)	−1.7 (1.2)	−1.2 (1.1)	−0.5 (−3.8 to 2.8; 0.77)
VC, L	0.14 (0.07)	0.11 (0.07)	0.03 (−0.16 to 0.23; 0.74)	0.09 (0.11)	−0.10 (0.07)	0.19 (−0.09 to 0.47; 0.17)
Others						
MIP, cmH_2_0	−8.2 (4.9)	−9.8 (3.2)**	1.6 (−10.1 to 13.4; 0.78)	−8.4 (5.6)	−4.3 (2.6)	−4.1 (−17.7 to 9.5; 0.52)
MEP, cmH_2_0	6.8 (4.4)	−3.8 (4.5)	10.7 (−2.2 to 23.5; 0.099)	15.6 (5.7)*	−4.3 (4.8)	19.9 (4.1 to 35.6; 0.015)
Food intake,kcal/day	122 (93)	−17 (86)	139 (−122 to 399; 0.28)	ND	ND	ND
MRC, score	−0.2 (0.2)	0.3 (0.3)	−0.4 (−1.2 to 0.3; 0.22)	−0.2 (0.2)	0.6 (0.3)	−0.7 (−1.4 to −0.1; 0.030)
Plasma NE, ng/ml	−0.063 (0.061)	−0.066 (0.067)	0.004 (−0.183 to 0.190; 0.97)	ND	ND	ND
IL-6 NE, pg/ml	1.52 (1.33)	0.08 (0.21)	1.44 (−1.35 to 4.22; 0.31)	ND	ND	ND
TNF-α, pg/ml	0.29 (0.15)	0.08 (0.06)	0.21 (−0.12 to 0.54; 0.21)	ND	ND	ND
Mean BP, mmHg	−13 (3)**	−3 (4)	−10 (−20 to 1; 0.061)	−2 (3)	4 (4)	−6 (−17 to 4; 0.20)
Body weight, kg	0.1 (0.3)	0.4 (0.3)	−0.3 (−1.2 to 0.7; 0.58)	0.8 (0.4)	0.4 (0.4)	0.4 (−0.7 to 1.4; 0.49)
Total lean mass, kg	0.2 (0.5)	0.5 (0.3)	−0.2 (−1.5 to 1.1; 0.73)	ND	ND	ND
Grip strength, kg	0.3 (0.9)	−0.0 (0.5)	0.3 (−1.7 to 2.3; 0.76)	1.1 (0.9)	2.5 (1.1)*	−1.5 (−4.4 to 1.4; 0.31)

Data are means (SE), or mean effect (95% CI; p value) unless otherwise indicated. BP = blood pressure; FEV_1_ = forced expiratory volume in one second; FVC = forced vital capacity; IL = interleukin; MEP = maximal expiratory pressure; MIP = maximal inspiratory pressure; MRC = medical research council; ND = not done; NE = norepinephrine; PFT = pulmonary function test; VC = vital capacity.

* p<0.05,

** p<0.01: change between pre-treatment and post-treatment within-group difference.

### Food Intake

Food intake was assessed as described previously [13].

### Respiratory and Peripheral Muscle Strength

The maximal inspiratory pressure (MIP) and maximal expiratory pressure (MEP) were measured as described previously [13]. Peripheral muscle strength was measured by the maximal voluntary handgrip maneuver as described previously [13].

### Dual-Energy X-ray Absorptiometry (DEXA)

All participating centers measured dual energy x-ray absorptiometry (DEXA) to assess the total body composition, including lean body mass. The measurements were performed with the subject lying in a supine position. As a general rule, a single expert from each center analyzed the scans from the corresponding center.

### Blood Samples and Analyses

Serum GH, serum insulin-like growth factor (IGF)-1, serum tumor necrosis factor α (TNF-α), serum interleukin-6 (IL-6), and plasma norepinephrine were measured as described previously [13]. Additional details are described online in Supplementary Methods S1.

### Sample Size

The study’s target accrual was 60 in the original protocol at the time of study design (see supporting information; Protocol S1). When 31 of the 33 randomized patients completed this study, we re-performed the power and sample size calculation, and confirmed that the number of patients that had completed the study exceeded the number necessary for the re-calculated sample size of 18. As a result, this trial ended prematurely. Because i) it is difficult to prolong hospitalization considering the current status of health care insurance in Japan, and ii) what constituted a clinically important change in 6-MWD after ghrelin treatment with PR was not known before the study ended; the sample size calculation was re-performed on the estimated effect of only ghrelin treatment for improving 6-MWD, which was based on information from the pilot study [13]. The resultant total sample size of 18 was finally used to provide the power (80%) to detect a mean difference of 60 m in 6-MWD with an estimated SD of 40 m using a two-sided alpha of 0.05, though the study’s target accrual stated in the original protocol was 60.

**Figure 3:**
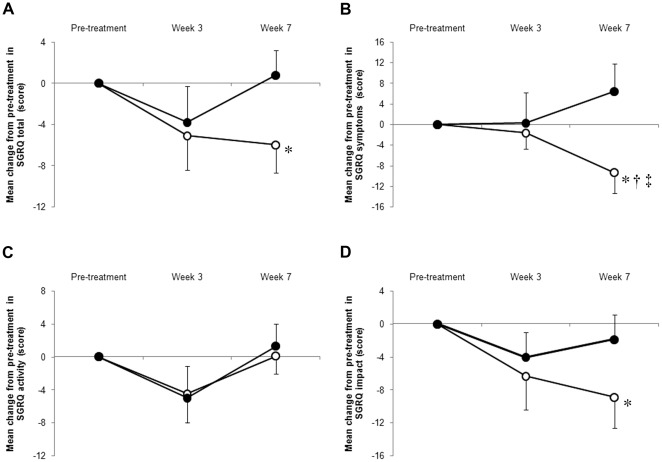
Change from pre-treatment in St. George Respiratory Questionnaire (SGRQ) scores over time. Open circles, ghrelin; closed circles, placebo. Data are presented as mean differences±SE. * p<0.05: change between pre- and post-treatment (within-group difference). ^†^ p<0.05: change between pre-treatment and post-treatment (between ghrelin and placebo group difference). ^‡^ p<0.05: time course effect of ghrelin versus placebo by repeated-measures ANOVA. At Week 3, marked improvements in SGRQ scores are not seen in both groups. However, SGRQ scores, especially SGRQ symptom scores, are significantly improved in the ghrelin group at Week 7. B) Repeated-measures ANOVA indicated significant time course effects of ghrelin versus placebo in SGRQ symptoms (F (2, 51) = 3.19, p = 0.049).

**Table 3:** Changes in pre-treatment scores of health-related quality of life during pulmonary rehabilitation with ghrelin or placebo

	At Week 3			At Week 7		
	Ghrelin,n = 14	Placebo,n = 15	Treatment effect(95% CI; p value)	Ghrelin,n = 14	Placebo,n = 13	Treatment effect(95% CI; p value)
SGRQ						
Total	−5.0 (3.2)	−3.9 (3.5)	−1.1 (−10.9 to 8.7; 0.83)	−6.0 (2.7)*	0.8 (2.4)	−6.8 (−14.4 to 0.7; 0.072)
Symptoms	−1.7 (3.0)	0.3 (5.9)	−1.9 (−16.2 to 12.3; 0.77)	−9.4 (4.0)*	6.4 (5.4)	−15.8 (−29.5 to −2.1; 0.026)
Activity	−4.5 (3.5)	−5.0 (3.9)	0.4 (−10.5 to 11.4; 0.94)	0.1 (2.2)	1.3 (2.7)	−1.2 (−8.3 to 5.9; 0.73)
Impacts	−6.3 (4.1)	−4.1 (3.1)	−2.2 (−12.6 to 8.2; 0.67)	−8.9 (3.7)*	−1.9 (3.0)	−7.0 (−16.9 to 2.9; 0.16)
SF-36						
Physicalfunctioning	4.6 (6.1)	0.3 (3.9)	4.3 (−10.0 to 18.5; 0.55)	3.1 (4.7)	−6.9 (4.9)	10.0 (−3.9 to 23.9; 0.15)
Role physical	−8.3 (6.9)	−4.6 (5.4)	−3.7 (−21.6 to 14.1; 0.67)	−12.0 (4.1)*	−22.6 (7.3)**	10.6 (−6.8 to 27.9; 0.22)
Bodily pain	−6.8 (5.3)	8.4 (6.4)	−15.2 (−33.0 to 2.6; 0.090)	−7.6 (6.5)	−3.8 (6.8)	−3.8 (−23.2 to 15.7; 0.69)
General health	−0.6 (4.5)	2.9 (5.2)	−3.5 (−17.9 to 11.0; 0.63)	0.5 (3.4)	5.8 (5.4)	−5.3 (−18.5 to 7.9; 0.41)
Vitality	5.7 (5.5)	7.8 (4.4)	−2.0 (−16.3 to 12.3; 0.77)	3.4 (4.8)	−2.9 (3.4)	6.2 (−5.9 to 18.4; 0.30)
Social functioning	−3.1 (9.5)	3.3 (7.2)	−6.5 (−30.5 to 17.6; 0.59)	−12.5 (8.1)	−2.9 (6.0)	−9.6 (−30.5 to 11.3; 0.35)
Role emotional	−13.9 (5.2)*	−9.5 (9.2)	−4.4 (−27.7 to 18.8; 0.68)	−19.9 (6.6)*	−16.0 (10.4)	−3.9 (−29.3 to 21.5; 0.76)
Mental health	0.4 (6.0)	3.7 (4.2)	−3.3 (−18.0 to 11.5; 0.65)	3.5 (3.3)	−8.2 (4.6)	11.7 (0.0 to 23.4; 0.050)

Data are means (SE), or mean effect (95% CI; p value) unless otherwise indicated. SGRQ = St. George Respiratory Questionnaire; SF 36 = short-Form 36.

* p<0.05,

** p<0.01: change between pre-treatment and post-treatment within-group difference.

### Statistical Analysis

All data are expressed as means±SD or SE unless otherwise indicated. Comparisons of baseline characteristics between the two groups were made by Fisher’s exact tests and Wilcoxon rank sum tests. Effects were examined once or twice; that is i) at Week 3 soon after 3-week treatment or ii) at Week 3 and Week 7 (i.e., 4 weeks after the completion of 3-week treatment). The results at Week 3 and Week 7, respectively, were compared with the pre-treatment within each group, and between the two groups using paired *t*-tests and unpaired *t-*tests, respectively. To assess the time course efficacy of ghrelin versus placebo, post-treatment data up to Week 7 were also assessed using a repeated-measures analysis of variance (ANOVA). A p value<0.05 was considered significant (SAS 9.1.3, SAS Institute Inc., Cary, NC, USA).

**Table 4:** Adverse events.

Event	Ghrelin, n = 18	Placebo, n = 15
Patients with at least 1 adverse event	12 (67)	5 (33)
Adverse events not considered study therapy-related		
Pneumonia	1 (6)	0 (0)
Depression	1 (6)	0 (0)
Infective enteritis	1 (6)	0 (0)
Lung cancer*	1 (6)	0 (0)
Hypercalcemia	0 (0)	1 (7)
Adverse events considered study therapy-related		
Stomach rumbling	3 (17)	2 (13)
Feeling of being warm	4 (22)	0 (0)
Feeling of hunger	2 (11)	2 (13)
Thirst	2 (11)	0 (0)
Slight liver dysfunction	1 (6)	0 (0)
Hypercholesterolemia	1 (6)	0 (0)
Hypoproteinemia	1 (6)	2 (13)

Values are presented as n (% of group). * One patient developed lung cancer 2 years and 9 months after study treatment.

## Results

Of the 33 randomized patients, 31completed the 3-week study; 2 patients in the ghrelin group discontinued study medications due to pneumonia and depression, respectively. Of the 31 patients who completed the randomized 3-week study, in the ghrelin group, one patient had infective enteritis after 3 weeks of medications, and one had low back pain due to lumbar spondylosis before and throughout the 3 weeks of medications. Two patients in the placebo group were lost to follow-up after the Week 3 measurements. Therefore, 29 patients (ghrelin, n = 14; placebo, n = 15) were included in the study analyses to ensure adequate efficacy evaluation using pre-protocol analysis. The mean BMI in the enrolled patients (n = 29) was very low (mean±SD, 18.3±2.1 kg/m^2^). The treatment groups were generally well-matched with regard to demographics and baseline characteristics (Table 1).

### Somatotropic Function

At pre-treatment, compared with placebo, a single administration of ghrelin markedly increased serum GH levels from baseline (mean change±SE: ghrelin group 46.4±6.2 ng/ml at the mean peak time (35 min) versus the placebo group 1.1±0.5 ng/ml at the mean peak time (55 min); between group p<0.0001), the effect of which was maintained at Week 3 (mean change±SE: ghrelin group 15.8±2.1 ng/ml at the mean peak time (30 min) versus the placebo group 0.4±0.2 ng/ml at the mean peak time (65 min); between group p<0.0001). Three-week ghrelin-PR combination treatment tended to increase serum IGF-1 levels (mean change±SE: 12±6 ng/ml, within-group p = 0.093).

### Exercise Tolerance and Gas Exchange Measurements

At both Week 3 and Week 7, there were no significant differences between the ghrelin and placebo groups in 6-MWD. In each group, at Week 3, a similar significant increase from pre-treatment in 6-MWD was observed (mean difference: ghrelin group +40 m, within group p = 0.033 versus placebo group +35 m, within group p = 0.013). The effect remained at Week 7 in the ghrelin group, whereas in the placebo group, the improvement in 6-MWD was reduced at Week 7 (mean difference: ghrelin group within group +47 m, p = 0.017 versus placebo group +18 m, within group p = 0.14) (Table 2 and Figure 2A). To assess the time course efficacy of ghrelin versus placebo in 6-MWD, a repeated-measures ANOVA was performed. There was no significant time course effect of ghrelin versus placebo in 6-MWD (F (2, 51) = 1.10, p = 0.34).

In the ghrelin group, the peak 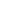 and 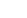/HR were significantly increased by 1.2 ml/kg/min and 0.5 ml/beats, respectively, from pre-treatment (within-group p = 0.021, p = 0.019, respectively) (Table 2). However, there was no significant difference between the two groups in the peak 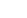 and 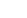/HR. In the ghrelin group, the ventilatory equivalents for oxygen (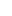
E/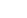) was relatively improved by −3.9 from pre-treatment (within group p = 0.060).

### HRQoL and MRC Measures

In both groups, there was no significant difference in each SGRQ score and MRC score between pre-treatment and at Week 3. At Week 7, there was a significant treatment effect between the two groups in SGRQ symptoms (between-group: p = 0.026, Table 3 and Figure 3B), and in the MRC score (between-group p = 0.030, Table 2 and Figure 2B). At Week 7, in the ghrelin group, SGRQ total was decreased by 6.0 from pre-treatment (within-group p = 0.046, between-group p = 0.072) (Table 3 and Figure 3A). Furthermore, there was a significant time course effect of ghrelin versus placebo in SGRQ symptoms (repeated-measures ANOVA, F (2, 51) = 3.19, p = 0.049, Figure 3B).

### Body Weight and Food Intake

In the ghrelin group, at Week 1, the relative increase in body weight was+0.42 kg (within group p = 0.092), which was reduced by Week 3 and followed by a re-increase at Week 7 (+0.8 kg, within group: p = 0.054). However there was no significant difference in body weight between the groups at each Week (Table 2). No affect on whole lean body mass from ghrelin was seen at Week 3 (Table 2). No significant increase from baseline in food intake was observed at Week 3 in both groups (Table 2).

### Respiratory and Peripheral Muscle Strength

In the ghrelin group, at Week 3, the post-treatment increase in respiratory muscle strength, as indicated by MEP and MIP, was not significantly different from that in the placebo group, but at Week 7, the mean increase from pre-treatment in MEP (+15.6 cmH_2_O) was significantly different from that in the placebo group (between group p = 0.015) (Table 2). Furthermore, there was a significant time course effect of ghrelin versus placebo in MEP (repeated-measures ANOVA, F (2, 51) = 4.17, p = 0.021, Figure 2C).

At Week 3 and Week 7, there was no significant treatment effect between the two groups in grip strength (Table 2).

### Pulmonary Function, Plasma Norepinephrine, and Other Hormone Levels

Ghrelin treatment did not significantly change any parameters of the pulmonary function tests, serum TNF-α, serum IL-6, or plasma norepinephrine at rest (Table 2).

### Safety

Throughout this trial, 67% of patients in the ghrelin group and 33% of patients in the placebo group reported 12 and 5 adverse events, respectively, but there was no significant difference between the groups (Table 4). In the ghrelin group, alanine aminotransferase increased to 41 IU/L in one patient (6%), and total cholesterol increased to 270 mg/dl in one patient (6%); both increases disappeared at Week 7. Two patients randomized to ghrelin discontinued as a result of adverse events: one because of bacterial pneumonia, and one because of depression, both of which were not considered related to ghrelin treatment. One patient randomized to ghrelin developed lung cancer 2 years and 9 months after the end of ghrelin administration, but this was judged by the efficacy and safety committee as not causally related to ghrelin treatment, considering the period of disease development and the incidence rate of lung cancer [19].

### **Discussion**

The present study is the first multicenter, randomized, double-blind, placebo-controlled study to assess the effect and safety of repeated ghrelin administration to very severe cachectic patients with COPD. The main results of this study can be summarized as follows. In the ghrelin group, single administration of ghrelin was accompanied by a significant increase in serum GH levels during 3-week treatment, and there was no significant difference in 6-MWD between ghrelin and placebo at Week 3 and at Week 7. With ghrelin, symptomatic improvements in SGRQ symptoms and MRC score were not obtained at Week 3, but significant differences between ghrelin and placebo were seen at Week 7. In the ghrelin group, no significant within-group improvement from pre-treatment was seen in respiratory muscle strength, as indicated by MEP and MIP, at Week 3, but there was a significant difference in MEP between ghrelin and placebo at Week 7. Repeated-measures ANOVA showed significant time course effects of ghrelin versus placebo in SGRQ symptoms and MEP. Finally, ghrelin treatment was well tolerated.

Ghrelin treatment may have beneficial, continuing effects after treatment on HRQoL and MRC measures in this population. Though this study was conducted to determine the effectiveness of ghrelin in cachectic COPD patients, considering a synergistic interaction between ghrelin and PR, the data of this study need to be interpreted with caution, because, especially in advanced stage patients, excessive exercise training may partially worsen the anabolic and catabolic balance [1], [20]. In the present study, which included patients with a lower exercise capacity and pulmonary function than those in the pilot study [13] and more cachectic patients than those in other studies on PR [21], the 6-MWD after 3-week PR in the placebo group was decreased in 3 (20%) of the 15 patients. Since 5 patients (33%) in the placebo group found the initial training work rate intolerable, the initial training work rate remained at its initial setting. In addition, at Week 3, outcome measurements showed no improvements with ghrelin compared with placebo. These findings may represent patients’ variable responses to PR, which might have an influence on the effects of ghrelin. Of note, however, there were significant treatment effects of ghrelin in both SGRQ symptoms and MRC score. In addition, the treatment tended to improve the total SGRQ score by more than 4 points; a clinically meaningful improvement. These effects were not observed soon after the 3 week-treatment, but were seen 4 weeks after treatment, maintaining the improvement obtained in 6-MWD at Week 3. Similarly, 4 weeks after treatment, the effect of ghrelin on respiratory muscle strength was confirmed, though it has been reported that GH alone does not increase strength in healthy elderly [22], [23], [24]. Furthermore, repeated-measures ANOVA indicated significant time course effects of ghrelin versus placebo in SGRQ symptoms and MEP. Our data suggest that improving of the respiratory muscle strength, the O_2_ pulse, and the ventilatory equivalents for oxygen may serve as a mechanism by which ghrelin-PR combination treatment improved symptoms, though further examination is needed to understand the precise mechanism. These findings suggest that repeated ghrelin administration may have beneficial, sustained effects after administration on symptoms through GH-dependent and/or -independent mechanisms.

Cachectic elderly patients with COPD who were given intravenous ghrelin showed a continuous increase of pulsatile GH secretion in the present study. There is evidence that insufficiency of sarcopenia-related hormones, such as GH and IGF-1, may contribute to cachexia [25], [26]. Observational studies in cachectic COPD patients have found decreased levels of these hormones [27], [28]. In the present study, despite significant increases in GH secretion levels throughout the 3-week treatment and respiratory muscle strength, ghrelin provided only a significant within-group increase in exercise performance, and a relative within-group increase in IGF-1 levels and body weight. Furthermore, ghrelin did not affect food intake, grip strength or plasma norepinephrine levels at rest in the present study. Although DEXA should be performed a greater number of times during the trial, at Week 3 ghrelin did not show any effects on whole lean body mass. Meanwhile, previous studies showed that ghrelin administration induced a positive energy balance and weight gain [8], increased food intake [9], [13], and decreased sympathetic nervous activity [10], [11], [13]. The discrepancy may be explained by the fact that the intensity of exercise training for some cachectic participants counteracted the effects of ghrelin, though lower extremity exercise training at higher intensity produces greater benefits than lower intensity training [4]. As one of the reasons, the patients treated with both ghrelin and exercise training gained at Week 1, which was not seen in the placebo group. However, this weight gain reduced by Week 3. At Week 7, the weight was regained (Table 2). The days of attending PR in the ghrelin group was negatively correlated with the increase in body weight from Week 3 to Week 7 (r = −0.710, p = 0.003). We speculate that the unintended excessive exercise permitted by ghrelin administration with antidepressant-like effects [29] might prevent the obtained results. Nevertheless, these findings suggest that clinical interventions with ghrelin may help cachectic COPD patients via inhibiting somatopause and regulating metabolic balance.

The participants in the present study tolerated daily administration of ghrelin for 3 weeks (Table 4); the most frequent ghrelin-related side effects were mild and similar to those of previous reports [13], [30], [31], as well as with those of GH administration by injection [22]. However, given that the previous studies of the responses of ghrelin in proliferation, including tumor development, have demonstrated conflicting findings [32], [33], [34], [35], more studies of the safety of ghrelin treatment are necessary before clinical application.

This study had some limitations. First, the number of participants was small, and few females were included in this trial. Second, the duration of the study was short. A more effective exercise training program, considering its intensity and frequencies, should have been conducted. Additional studies are needed to evaluate a more suitable regimen of ghrelin-PR.

In conclusion, ghrelin administration provided sustained improvements in symptoms and respiratory strength in cachectic COPD patients. Development of ghrelin administration methods may offer potential advantages over the currently approved treatment options for COPD. The lack of a significant between-group difference in exercise tolerance may result from the exercise training program conducted as the combination therapy. Careful examination is needed to develop more effective administration methods of ghrelin and combination therapy with ghrelin.

## Supporting Information

Methods S1 (DOC)

Protocol S1 (DOC)

Checklist S1 (DOC)

## References

[1] Wagner PD (2008). Possible mechanisms underlying the development of cachexia in COPD.. Eur Respir J.

[2] Wilson DO, Rogers RM, Wright EC, Anthonisen NR (1989). Body weight in chronic obstructive pulmonary disease. The National Institutes of Health Intermittent Positive-Pressure Breathing Trial.. Am Rev Respir Dis.

[3] Burdet L, de Muralt B, Schutz Y, Pichard C, Fitting JW (1997). Administration of growth hormone to underweight patients with chronic obstructive pulmonary disease. A prospective, randomized, controlled study.. Am J Respir Crit Care Med.

[4] Ries AL, Bauldoff GS, Carlin BW, Casaburi R, Emery CF (2007). Pulmonary Rehabilitation: Joint ACCP/AACVPR Evidence-Based Clinical Practice Guidelines.. Chest.

[5] Zinna EM, Yarasheski KE (2003). Exercise treatment to counteract protein wasting of chronic diseases.. Curr Opin Clin Nutr Metab Care.

[6] Zaloga GP (2005). Ghrelin, diet, and pulmonary function.. Chest.

[7] Kojima M, Hosoda H, Date Y, Nakazato M, Matsuo H (1999). Ghrelin is a growth-hormone-releasing acylated peptide from stomach.. Nature.

[8] Tschop M, Smiley DL, Heiman ML (2000). Ghrelin induces adiposity in rodents.. Nature.

[9] Nakazato M, Murakami N, Date Y, Kojima M, Matsuo H (2001). A role for ghrelin in the central regulation of feeding.. Nature.

[10] Matsumura K, Tsuchihashi T, Fujii K, Abe I, Iida M (2002). Central ghrelin modulates sympathetic activity in conscious rabbits.. Hypertension.

[11] Nagaya N, Kojima M, Uematsu M, Yamagishi M, Hosoda H (2001). Hemodynamic and hormonal effects of human ghrelin in healthy volunteers.. Am J Physiol Regul Integr Comp Physiol.

[12] Itoh T, Nagaya N, Yoshikawa M, Fukuoka A, Takenaka H (2004). Elevated plasma ghrelin level in underweight patients with chronic obstructive pulmonary disease.. Am J Respir Crit Care Med.

[13] Nagaya N, Itoh T, Murakami S, Oya H, Uematsu M (2005). Treatment of cachexia with ghrelin in patients with COPD.. Chest.

[14] Jones PW, Quirk FH, Baveystock CM, Littlejohns P (1992). A self-complete measure of health status for chronic airflow limitation. The St. George’s Respiratory Questionnaire.. Am Rev Respir Dis.

[15] Ware JE, Sherbourne CD (1992). The MOS 36-item short-form health survey (SF-36). I. Conceptual framework and item selection.. Med Care.

[16] Fukuhara S, Bito S, Green J, Hsiao A, Kurokawa K (1998). Translation, adaptation, and validation of the SF-36 Health Survey for use in Japan.. J Clin Epidemiol.

[17] Fukuhara S, Ware JE, Kosinski M, Wada S, Gandek B (1998). Psychometric and clinical tests of validity of the Japanese SF-36 Health Survey.. J Clin Epidemiol.

[18] Fletcher CM, Elmes PC, Fairbairn AS, Wood CH (1959). The significance of respiratory symptoms and the diagnosis of chronic bronchitis in a working population.. Br Med J.

[19] Calverley PM, Spencer S, Willits L, Burge PS, Jones PW (2003). Withdrawal from treatment as an outcome in the ISOLDE study of COPD.. Chest.

[20] Rabinovich RA, Ardite E, Mayer AM, Polo MF, Vilaro J (2006). Training depletes muscle glutathione in patients with chronic obstructive pulmonary disease and low body mass index.. Respiration.

[21] Puhan MA, Schunemann HJ, Frey M, Scharplatz M, Bachmann LM (2005). How should COPD patients exercise during respiratory rehabilitation? Comparison of exercise modalities and intensities to treat skeletal muscle dysfunction.. Thorax.

[22] Liu H, Bravata DM, Olkin I, Nayak S, Roberts B (2007). Systematic review: the safety and efficacy of growth hormone in the healthy elderly.. Ann Intern Med.

[23] Papadakis MA, Grady D, Black D, Tierney MJ, Gooding GA (1996). Growth hormone replacement in healthy older men improves body composition but not functional ability.. Ann Intern Med.

[24] Blackman MR, Sorkin JD, Munzer T, Bellantoni MF, Busby-Whitehead J (2002). Growth hormone and sex steroid administration in healthy aged women and men: a randomized controlled trial.. JAMA.

[25] Anawalt BD, Merriam GR (2001). Neuroendocrine aging in men. Andropause and somatopause.. Endocrinol Metab Clin North Am.

[26] Lamberts SW, van den Beld AW, van der Lely AJ (1997). The endocrinology of aging.. Science.

[27] Creutzberg EC, Wouters EF, Mostert R, Pluymers RJ, Schols AM (2003). A role for anabolic steroids in the rehabilitation of patients with COPD? A double-blind, placebo-controlled, randomized trial.. Chest.

[28] Koehler F, Doehner W, Hoernig S, Witt C, Anker SD (2007). Anorexia in chronic obstructive pulmonary disease–association to cachexia and hormonal derangement.. Int J Cardiol.

[29] Lutter M, Elmquist J (2009). Depression and metabolism: linking changes in leptin and ghrelin to mood.. F1000 Biol Rep.

[30] Akamizu T, Takaya K, Irako T, Hosoda H, Teramukai S (2004). Pharmacokinetics, safety, and endocrine and appetite effects of ghrelin administration in young healthy subjects.. Eur J Endocrinol.

[31] Nagaya N, Moriya J, Yasumura Y, Uematsu M, Ono F (2004). Effects of ghrelin administration on left ventricular function, exercise capacity, and muscle wasting in patients with chronic heart failure.. Circulation.

[32] Cassoni P, Allia E, Marrocco T, Ghe C, Ghigo E (2006). Ghrelin and cortistatin in lung cancer: expression of peptides and related receptors in human primary tumors and in vitro effect on the H345 small cell carcinoma cell line.. J Endocrinol Invest.

[33] Belloni AS, Macchi C, Rebuffat P, Conconi MT, Malendowicz LK (2004). Effect of ghrelin on the apoptotic deletion rate of different types of cells cultured in vitro.. Int J Mol Med.

[34] Ghe C, Cassoni P, Catapano F, Marrocco T, Deghenghi R (2002). The antiproliferative effect of synthetic peptidyl GH secretagogues in human CALU-1 lung carcinoma cells.. Endocrinology.

[35] Murata M, Okimura Y, Iida K, Matsumoto M, Sowa H (2002). Ghrelin modulates the downstream molecules of insulin signaling in hepatoma cells.. J Biol Chem.

