# Supplementary material for: Ghrelin Treatment of Cachectic Patients with Chronic Obstructive Pulmonary Disease: A Multicenter, Randomized, Double-Blind, Placebo-Controlled Trial
Source: PLoS One. 2012 May 1;7(5):e35708. doi: 10.1371/journal.pone.0035708 (PMC3341383; doi:10.1371/journal.pone.0035708)
Supplement: Methods S1 — (DOC) [file pone.0035708.s001.doc]

**Ghrelin Treatment of Cachectic Patients with Chronic Obstructive Pulmonary Disease: a multicenter, randomized, double-blind, placebo-controlled trial**

Keisuke Miki, MD, PhD; Ryoji Maekura, MD, PhD; Noritoshi Nagaya, MD, PhD; Masamitsu Nakazato, MD, PhD; Hiroshi Kimura, MD, PhD; Shinsuke Murakami, MD; Shunsuke Ohnishi MD, PhD; Toru Hiraga, MD, PhD; Mari Miki, MD, PhD; Seigo Kitada, MD, PhD; Kenji Yoshimura MD; Yoshitaka Tateishi, MD, PhD; Yasuji Arimura MD, MPH; Nobuhiro Matsumoto, MD, PhD; Masanori Yoshikawa, MD, PhD; Kenichi Yamahara, MD, PhD; Kenji Kangawa, PhD

**Supplementary Methods S1**

**Pulmonary rehabilitation (PR)**

The PR program consisted of education, breathing control techniques, and exercise training on an inpatient basis. Exercise training was conducted in 3 sets daily, every weekday for 3 weeks (i.e. 15 days) at high-intensity targets using electromechanically braked cycle ergometers. The initial exercise level of each set was set for 6 min at the work rate corresponding to 60% of the peak Combino_2_ achieved on the baseline cardiopulmonary exercise testing (CPET). As tolerated by the subject, the exercise duration was initially increased to 10 min. After that, the training work rate was increased by 5 W and then extended to the work rate corresponding to 80% of the baseline peak Combino_2_. If the subject found the set intolerable, it was reduced to its previous setting. Supplemental oxygen was used if necessary to maintain an oxygen saturation > 90% during exercise training. In this program, both occupational therapy such as exercise training in their activities of daily living and PR on an outpatient basis were not conducted.

**Blood samples and analyses**

Blood sample were taken after 30-min bed rest in the morning following an overnight fast. Serum GH and insulin-like growth factor (IGF)-1levels were measured by immunoradiometric assay as described previously. ^E1^ Serum tumour necrosis factor α (TNF-α) and interleukin-6 (IL-6) concentrations were measured by enzyme immunoassay as described previously. ^E1^ Plasma norepinephrine concentration was measured by high-performance liquid chromatography as described previously. ^E1^

E1.　Nagaya N, Itoh T, Murakami S, Oya H, Uematsu M, et al. (2005) Treatment of cachexia with ghrelin in patients with COPD. Chest 128: 1187-1193.
